# Membrane Tethering Complexes in the Endosomal System

**DOI:** 10.3389/fcell.2016.00035

**Published:** 2016-05-09

**Authors:** Anne Spang

**Affiliations:** Biozentrum, Growth & Development, University of BaselBasel, Switzerland

**Keywords:** endocytosis, exocytosis, membrane contact sites, membrane fusion, recycling, Golgi, vesicle trafficking

## Abstract

Vesicles that are generated by endocytic events at the plasma membrane are destined to early endosomes. A prerequisite for proper fusion is the tethering of two membrane entities. Tethering of vesicles to early endosomes is mediated by the class C core vacuole/endosome tethering (CORVET) complex, while fusion of late endosomes with lysosomes depends on the homotypic fusion and vacuole protein sorting (HOPS) complex. Recycling through the trans-Golgi network (TGN) and to the plasma membrane is facilitated by the Golgi associated retrograde protein (GARP) and endosome-associated recycling protein (EARP) complexes, respectively. However, there are other tethering functions in the endosomal system as there are multiple pathways through which proteins can be delivered from endosomes to either the TGN or the plasma membrane. Furthermore, proteins that may be part of novel tethering complexes have been recently identified. Thus, it is likely that more tethering factors exist. In this review, I will provide an overview of different tethering complexes of the endosomal system and discuss how they may provide specificity in membrane traffic.

## Introduction

Most cellular membrane-bound organelles communicate with each other either through vesicles that shuttle between membrane entities or via direct contacts between organellar membranes. Vesicles or transport containers that are formed at one compartment will have to find the correct target compartment to deliver their content. This regulated vectorial transport provides the basis of intracellular membrane transport. The recognition system at the donor compartment is multilayered to ensure specificity. Already at the initiation stage of vesicle formation, SNARE proteins are included into the nascent vesicle through direct interactions with a small GTPase of the Sar/Arf family and coat components (Springer et al., 1999; Spang, 2002, 2008; Bonifacino and Glick, 2004). The function of SNAREs is twofold. First, they recognize complementary SNAREs on the donor membrane and second their engagement into a tertiary trans-SNARE complex brings vesicle and acceptor compartment lipid bilayers in such close opposition that membrane fusion is promoted. The SNAREs however are small molecules and are covered by coat proteins on the transport vesicle. Hence, they cannot reach far into the cytoplasm and only act very late in the vesicle-target membrane recognition process. This begs for another earlier detection system; such a system is provided by tethers. Tethers are extended proteins or protein complexes present mostly on the target membrane sampling the environment for presence of the correct vesicles. Upon successful contact, they bring vesicles closer to the target membrane (Yu and Hughson, 2010). The recognition process is helped by small GTPases of the Rab family. This long- and short-range double identification process ensures correct targeting of intracellular vesicles and improves the fidelity of the entire process. The same mechanism also operates during membrane fusion of organelles such as homotypic fusion of endosomes in metazoans and the vacuole in yeast. Besides tethers that are involved in membrane fusion, other types of tethering molecules exist acting as a linker between membranes, such as the ERMES complex in yeast, which connects the ER and mitochondria, or ER-plasma membrane tethers (Phillips and Voeltz, 2016). Although, these tethers also bring membranes into close proximity and promote the exchange of lipids between organelles, fusion is not the end result of these interactions. In this review, I will concentrate on tethers involved in membrane fusion in the endocytic pathway.

Endocytosis refers to the process by which cells take up molecules from the extracellular space and internalize plasma membrane proteins. Endocytic carriers formed at the plasma membrane are subsequently transported to early endosomes with which they fuse. In addition, early endosomes can fuse with each other. Following an ill-characterized switch, early endosomes can no longer accept plasma membrane derived carriers, and sorting of accumulated material occurs in the now called sorting endosome (Huotari and Helenius, 2011). Most of the endocytosed material is immediately recycled back to the plasma membrane, while the remainder will either be transported to the trans-Golgi network (TGN) or remain in the endosome, which eventually fuses with a lysosome resulting in the degradation of endocytosed material. Endosomes are also points of intersection between exocytic and endocytic routes: vesicles derived from the TGN can fuse with early and late endosomes. Additionally, while secretory vesicles use the exocyst complex as a tether for fusion with the plasma membrane, other entities such as recycling endosomes may depend on a different tether. The existence of an alternative tether at the plasma membrane is likely because exocyst is restricted to the baso-lateral membrane in polarized epithelial cells, whereas the tether at the apical membrane remains elusive (Lipschutz et al., 2000; Matern et al., 2001; Mukherjee et al., 2014). Thus, also in the endocytic pathway specific tethers must exist that control the fusion of membranes.

Our knowledge on different tethers in the endosomal system is still quite limited. However, a picture emerges in which modular protein complexes assemble on membranes to act as specific tethers. Here, I will provide an overview on different endocytic tethers and discuss what we might still be missing.

## Composition and function of the HOPS complex

**Figure 1:**
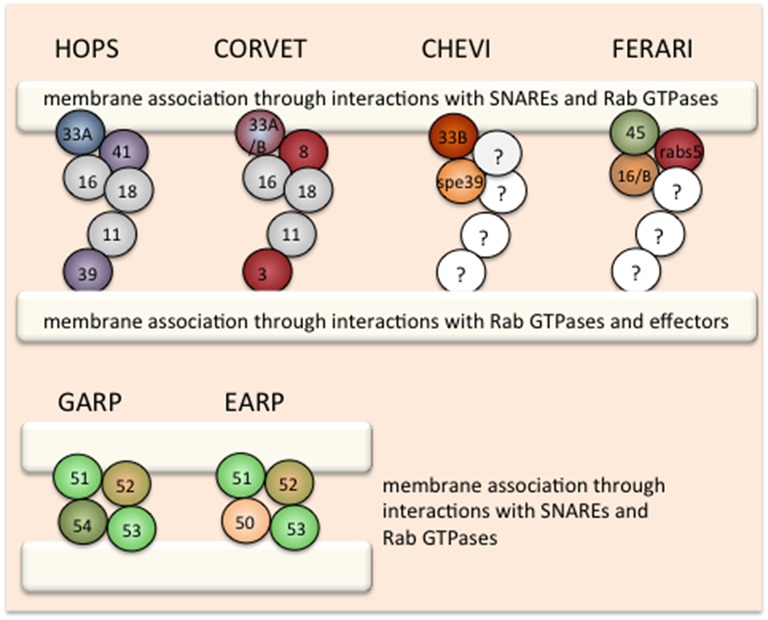
**Organization of tethering complexes in the endosomal system**. Graphical display of the HOPS and CORVET family of tethering complexes and the GARP/EARP complexes. The Rab interaction module is highlighted in each complex: blue: Rab7, red: Rab5, green/orange: Rab6/Rab4. The SM proteins in the HOPS and CORVET family are also marked with specific colors. In GARP the localizing subunit is colored in green and in EARP in orange. Membranes are indicated by white bars.

The first tether found in the endocytic system was the homotypic fusion and vacuole protein sorting (HOPS) complex (Seals et al., 2000; Wurmser et al., 2000). Initially identified in yeast through biochemical analysis, HOPS was mostly appreciated for its function in promoting homotypic fusion of vacuoles as part of their inheritance cycle. Soon it became clear, however, that fusion of late endosomes to the vacuole is also dependent on HOPS (Peterson and Emr, 2001; Bugnicourt et al., 2004). The HOPS complex consists of the core proteins Vps11, Vps16, and Vps18, the Ypt7/Rab7 interacting subunits Vps39 and Vps41 as well as the Sec1/Munc18 family (SM) protein Vps33 (Figure 1). The HOPS members were identified also in genetic screens for vacuolar transport deficiency (Bankaitis et al., 1986; Rothman and Stevens, 1986). The core components and the Vps33 belong to the class C mutants, in which coherent vacuoles were lacking (Banta et al., 1988), whereas *vps39* and *vps41* mutants showed a somewhat weaker phenotype in which vacuoles were fragmented. Thus, even though the HOPS complex is supposed to act as a complex, deletions of the individual components have different phenotypes, suggesting that the core components and the SM protein must have additional functions in the cell.

HOPS maintains two business ends related to its function. Vps39 and Vps41 recognize Ypt7/Rab7 and thus provide the link to the organelle with which fusion should be initiated (Wurmser et al., 2000; Plemel et al., 2011). Whether Vps39 and Vps41 recognize the same membrane or whether each of the subunits contact either of the two fusion partners is still not entirely clear. Vps39 bound to HOPS had initially been proposed to act as Ypt7 guanine nucleotide exchange factor (Ypt7-GEF; Wurmser et al., 2000). This notion has been challenged later, in that the Mon1-Ccz1 complex was shown to have GEF activity for Ypt7/Rab7, and it was suggested that HOPS might act as a buffer for the activated Ypt7-GTP (Nordmann et al., 2010). In this scenario, Vps39 would act as a potential GEF recruiter through direct binding to the GEF. Vps33 is a SNARE master regulating the membrane fusion process by recognizing trans-SNARE complexes and ensuring that only the correct SNARE assembly is allowed to proceed to promote membrane fusion (Subramanian et al., 2004; Lobingier and Merz, 2012; Lobingier et al., 2014; Baker et al., 2015). Thus, HOPS provides partner recognition through the Rab interactors Vps39 and Vps41 and proof-reading activity through the SM protein Vps33 to support membrane fusion at the yeast vacuole. Although most of the studies pertaining HOPS have been performed in yeast, there is ample evidence from experiments performed in *Caenorhabditis elegans*, Drosophila, zebrafish, and mammalian cells to ensure that HOPS functions are conserved and that HOPS act as a tether at lysosomes for different membrane entities including autophagosomes (Sevrioukov et al., 2005; Schonthaler et al., 2008; Jiang et al., 2014; Perini et al., 2014; Solinger and Spang, 2014; van Der Kant et al., 2015; Wartosch et al., 2015).

Structural studies revealed the architecture of the HOPS complex, and crystal structures are available for Vps16 in complex with Vps33 (Brocker et al., 2012; Baker et al., 2013). The HOPS complex has a club-like shape in which Vps39 is at the handle end and Vps41 in close proximity to Vps16 and Vps33 form the bulgy part. These ends are connected via Vps11 and Vps18 (Figure 1). Initially it was surprising that the Rab-interacting proteins—Vps39 and Vps41—are not in close proximity, indicating that they individually interact with Ypt7/Rab7. This might indicate that HOPS promotes fusion only of organelles that both carry Ypt7–Rab7 on their surface. This notion is supported by recent findings showing that Ypt7/Rab7 is required on both membranes for HOPS-dependent tethering using an *in vitro* proteoliposome reconstitution system (Ho and Stroupe, 2015). The *in vivo* demonstration, however, is still lacking.

## Composition and function of the CORVET complex

**Figure 2:**
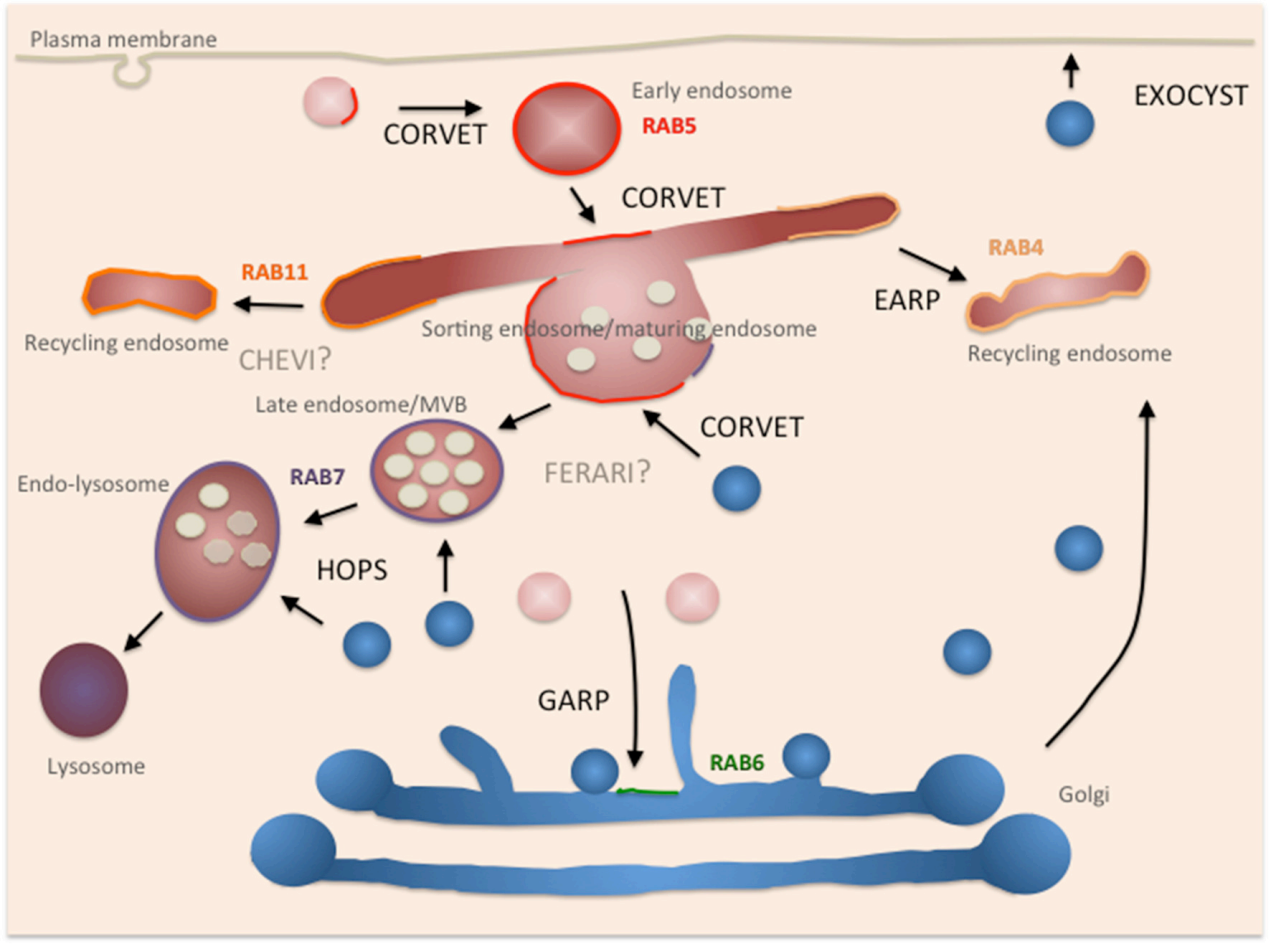
**Depiction of different tethers in the endosomal system and where they act/potentially act in the cell**. Secretory pathway components are depicted in blue, endocytic pathway components in red. Rabs are assigned to the appropriate compartment. Color code: Rab5: red, Rab7: blue, Rab11: orange, Rab4: ocher, Rab6: green.

The highly related class C core vacuole/endosome tethering (CORVET) complex is thought to act upstream of HOPS in the endocytic pathway (Peplowska et al., 2007). In yeast, the core subunits and the SM protein are identical to those found in HOPS, yet the Rab interacting subunits are different: Vps3 and Vps8 replace Vps39 and Vps41 and thereby change the Rab-interacting specificity to Vps21/Rab5 (Peplowska et al., 2007) (Figure 1). In analogy to HOPS, CORVET should tether Vps21/Rab5-positive membranes. Therefore, CORVET localizes to early endosomal membranes and potentially also to freshly generated endocytic carriers and macropinosomes (Figure 2). The identification of the CORVET complex also provides an explanation why the deletion phenotypes in yeast are more severe for the core and SM proteins than for Vps39 and Vps41. Considering the conservation of important pathways from yeast to man, it is not surprising that CORVET and its function as a tether on early endosomes are also conserved (Solinger and Spang, 2013, 2014; Perini et al., 2014; van Der Kant et al., 2015). Of consideration though is the requirement of the SM subunit Vps33 to mediate specificity in the fusion process, as the trans-SNARE complex on the vacuole/lysosome is not identical to the one on early endosomes. Thus, either Vps33 must be able to recognize two very distinct complexes or another regulatory protein providing specificity must exist. Alternatively, since early endosomes are also sorting stations, wrongly delivered cargo is sent back to its compartment of origin or diverted into different pathways and hence fusion specificity at this organelle is less important. This kind of backup system through alternative routes and partial redundancy of the delivery systems contribute to the robustness of the trafficking system but obviously complicates its analysis.

In metazoans, two Vps33 proteins are present, and hence CORVET and HOPS may each contain a different SM protein. While genetic evidence in *C. elegans* suggests this to be the case, biochemical analysis from mammalian tissue culture cells found the same Vps33 isoform (Vps33A) in both HOPS and CORVET complexes (Perini et al., 2014; Solinger and Spang, 2014; van Der Kant et al., 2015; Wartosch et al., 2015). These differences could be related to the experimental system and the evolutionary conservation of the SM proteins. In fact evolutionary analysis suggests that the *C. elegans* isoforms have diverged longer ago and are hence more different than the mammalian ones (Solinger and Spang, 2013). In addition, SM protein VPS-33.2 in *C. elegans* CORVET can be exchanged for VPS-33.1 under certain conditions in the gut epithelium (Solinger and Spang, 2014). Hence, it is conceivable that two different CORVET complexes exist, depending on the cell type and the flux of material through the endocytic pathway. Another peculiarity is that all HOPS and CORVET components are essential for development and survival in *C. elegans*, with one exception: The core component VPS-18. The *vps-18(tm1125)* null mutant is temperature-sensitive, but viable (http://www.wormbase.org/species/c_elegans/gene/WBGene00021058). Thus, another protein should exist taking over VPS-18 function. Intriguingly, no homolog for VPS-18 has been described to date. Structural homologs, however, may have a very different primary sequence.

## Other HOPS and CORVET-like complexes in the endosomal system

Other HOPS and CORVET-like complexes may differ in their Rab interacting module or the SM protein. In yeast, it has been suggested that HOPS and CORVET could be interconverted through intermediate, mixed complexes (Peplowska et al., 2007). It is also conceivable that exchanges between the SM proteins may occur in the tethering complex. This notion would be consistent with observations in *C. elegans* and may explain how CORVET can affect different tethering steps in mammalian cells (Perini et al., 2014). In addition, in metazoans, two Vps16 homologs, Vps16 and VIPAR/SPE-39/fob/Vps16B, are present. The current consensus is that Vps16 is present in both HOPS and CORVET, while SPE-39 appears to form a complex with Vps33B (VPS-33.2; van Der Kant et al., 2015). The function of the latter complex remains elusive. Also whether the Spe39-Vps33B complex functions as a dimer or other core components and a Rab interacting module are required for its function remains to be established. However, it is tempting to speculate that this is another HOPS and CORVET-like complex for which we propose the name CHEVI (class C Homologs in Endosome-Vesicle Interaction; Figure 1). Recently a retrograde transport pathway from the vacuole to endosomes has been proposed in yeast (Li et al., 2015), potentially requiring a specific tether. As such, CHEVI may function on endosomes to accept incoming vesicles from the TGN or the lysosome.

Unlike HOPS and CORVET, the SM protein is not an intrinsic component of most other tethers. Hence, there is a possibility that yet other types of complexes exist that carry Vps45, the third SM protein in the endosomal system. Vps16(B) and Vac1/rabenosyn5 have been identified as non-SNARE binding partners of Vps45 (Peterson et al., 1999; Nielsen et al., 2000; Kim et al., 2003; Gengyo-Ando et al., 2007). This tethering complex might represent a minimal HOPS and CORVET-like complex, as Vac1/rabenosyn5 mediates the interaction between Vps21/Rab5 and PI3P lipids (Peterson et al., 1999; Nielsen et al., 2000) and has been proposed to act at endosomes promoting recycling and retrograde transport to the TGN in a retromer-dependent manner (Rahajeng et al., 2010). Like HOPS and CORVET this complex contains a Rab interaction module and a SNARE proof-reading element. Hence, we propose the name FERARI (Factors for Endosome Recycling And Retromer Interactions; Figure 1). It is conceivable that even more complexes exist as VPS-33.1 potentially interacts with SPE-39 *in vitro* (Zhu et al., 2009).

## Moonlighting functions of HOPS components

Understanding the HOPS and CORVET tethers is further complicated through their assembly on membranes. At least for the HOPS complex, an assembly pathway has been postulated in which Arl8 first recruits Vps41 and this complex then catalyzes the binding of the other complex members (Khatter et al., 2015). Furthermore, the idea of an assembly pathway opens the possibility that complex members also have moonlighting functions in the cell, independent of tethering. In fact Vps41 was found to polymerize on AP-3-dependent transport carriers (Asensio et al., 2013; Pols et al., 2013). Additionally, Vps41 appeared to bind to caspase 8, and its overexpression promoted Fas-induced apoptosis (Wang et al., 2013). However, whether this latter function is indeed HOPS-independent remains to be determined, as no other HOPS components were tested.

Similarly to Vps41, Vps39 has at least one HOPS-independent function. Vps39 is part of mitochondrial-vacuolar contact sites, which allows the transport of phospholipids (Elbaz-Alon et al., 2014; Honscher et al., 2014). The precise nature of contact site tethers and the potential function of Ypt7/Rab7 in this process are not yet known. However, given these moonlighting functions of the Rab interaction module, it is conceivable that the CORVET-specific Rab interactors may also take over additional roles in the cell.

## EARP and GARP: Same principle, different components

Proteins that are not sent down to the lysosome or back to the plasma membrane may reach the TGN, where the Golgi associated retrograde protein (GARP) tethering complex awaits them. From the TGN, the other major sorting station of the cell, cargoes can reach the plasma membrane, endosomes, lysosomes, or even be transported back to the ER (Cho et al., 2012). Similar to the complexes described above, GARP consists of multiple subunits and interacts with a Rab protein (Ypt6/Rab6; Bonifacino and Hierro, 2011). However, unlike the HOPS and CORVET, no SM protein is part of the GARP complex. GARP belongs to the class of CATCHR tethering complexes of multiple subunit tethers to which the DSL complex, exocyst, COG also belong (Yu and Hughson, 2010; Spang, 2012). Interestingly, recently another tethering complex at endosomes has been described, endosome-associated recycling protein (EARP), which differs from GARP only in one subunit (Schindler et al., 2015). The exchange of the Vps54 subunit in GARP to syndetin/Vps50/VESA-1 in EARP causes localization of the complex to Rab4-positive recycling endosomes, which promote constitutive recycling of proteins such as the transferrin-receptor.

EARP does not interact with plasma membrane SNAREs, and hence the question remains, what membranes are tethered by EARP. In addition to EARP's localization to recycling endosomes, it is equally conceivable that EARP is present on sorting endosomes because co-localization of Rab5 and Rab11 with EARP were also observed (Schindler et al., 2015).

Nevertheless, conversion from HOPS to CORVET also causes a shift in localization from late endosomes/lysosomes to early endosomes. However, the correlation is not entirely perfect. While in CORVET and HOPS exchange of the subunit also changes the preference for Rab binding, this is less clear in GARP/EARP. The Ypt6/Rab6 binding protein Vps52 is part of both complexes. It is conceivable that Vps52 can interact with Rab4 and Rab6, dependent on its localization.

## Outlook and conclusion

A lot of open questions remain; below are a few to consider. Do we know the identity of all tethers in the endosomal system? Likely not, In particular the function of the orphan CHEVI complex needs to be established and the missing interaction partners for both CHEVI and FERARI must be identified. Since it is likely that the individual subunits of the tethers are not forming very stable complexes in the cytoplasm, we may not be able to detect easily other complexes by biochemical means. Hence, we will also require help from genetics. The combination of both will be instrumental in elucidating the identity and function of additional tethers in the endosomal system and beyond.

Is there exchange and conversion from one complex to the next? For example, following CORVET recruitment to early endosomes, does the core remain and the other subunits are turned over while new ones recruited to build HOPS? Likewise, does GARP reach the recycling endosome and is converted there into EARP? Alternatively, individual complex assembly may occur on membranes according to demand.

What can we make of the moonlighting functions of tether components? The action of Vps39 as a tether in organellar contact sites is very intriguing and it is conceivable that the cell reuses successful units in different contexts.

## Author contributions

The author confirms being the sole contributor of this work and approved it for publication.

### Conflict of interest statement

The author declares that the research was conducted in the absence of any commercial or financial relationships that could be construed as a potential conflict of interest.
